# Transcriptomic Profiling of Subcutaneous Backfat in Castrated and Intact Alentejano Pigs Finished Outdoors with Commercial and Fiber-Rich Diets

**DOI:** 10.3390/genes14091722

**Published:** 2023-08-29

**Authors:** Nicolás Garrido, André Albuquerque, Rui Charneca, Filipa Costa, Carla Marmelo, Amélia Ramos, Luísa Martin, José Manuel Martins

**Affiliations:** 1 ECO-PIG Consortium, Z.I. Catraia, Ap. 50, 3441-131 Santa Comba Dão, Portugal; nicolas.osa@uevora.pt (N.G.); andrealb@uevora.pt (A.A.); rmcc@uevora.pt (R.C.); lipaaa.costa@gmail.com (F.C.); carla.marmelo@racoessantiago.pt (C.M.); ameliaramos@esac.pt (A.R.); luisam@esac.pt (L.M.); 2 MED—Mediterranean Institute for Agriculture, Environment and Development & CHANGE—Global Change and Sustainability Institute, Universidade de Évora, Pólo da Mitra, Ap. 94, 7006-554 Évora, Portugal; 3 MED & CHANGE, Departamento de Zootecnia, ECT, Universidade de Évora, Pólo da Mitra, Ap. 94, 7006-554 Évora, Portugal; 4 Departamento de Ciências Agrárias e Tecnologias, Escola Superior Agrária de Coimbra, Bencanta, 3045-601 Coimbra, Portugal

**Keywords:** fat transcriptome, heavy pigs, fiber diet, boar taint, castration

## Abstract

In this work, we studied the backfat transcriptome of surgically castrated (C), intact (I) and intact fed an experimental diet (IE) outdoor-reared male Alentejano (AL) pigs. The experimental diet was a high-fiber diet with locally produced legumes and by-products associated with a boar taint reduction effect. At slaughter (~160 kg), backfat samples were collected for total RNA sequencing. Intact pigs presented leaner carcasses, more total collagen, and more unsaturated intramuscular fat content than C animals. A total of 2726 differentially expressed genes (DEGs, |log_2_ FC|> 0.58, q < 0.05) were identified between C and I with overexpressed genes related to muscular activity (*MYH1*, *ACTA1*) or collagen metabolism (*COL1A1*, *COL1A2*) in I pigs. Between C and IE, 1639 DEGs of genes involved in lipidic metabolism (*LEP*, *ME1*, *FABP4*, *ELOVL6*) were overexpressed in C. Finally, only 28 DEGs were determined between I and IE. Clustering results indicated a drastic influence of the testis in the transcriptome of subcutaneous fat of AL pigs, while the diet had a marginal effect. Diet can reduce stress by increasing satiety in animals, and could have induced an increase of skatole degradation due to the higher expression of the CYP2A19 gene in the IE group.

## 1. Introduction

The Alentejano (AL) pig is an autochthonous breed from the southern region of Portugal genetically similar to the Iberian pig [1] that belongs to the Mediterranean group of breeds descendant from the primitive *Sus scrofa mediterraneus* [2]. AL pigs are characterized by their light bone structure, black color, short and thin extremities and energetic nature, with slow growth rates and precocious lipogenic activity [3]. They are traditionally reared outdoors, are well adapted to the environmental conditions, and are slaughtered at heavy body weights (150 to 160 kg) for their high-quality manufactured dry-cured products [4]. These free-range systems are becoming increasingly popular as consumers’ awareness and environmental or animal welfare regulations change. However, one animal welfare regulation that is being discussed is the surgical castration of pigs, with stakeholders within the European Union signing a voluntary declaration to end this procedure [5]. This declaration may jeopardize the current system of AL/Iberian farms, where surgical castration is common practice.

Surgical castration of male pigs is a practice that has been performed for a long time to prevent unwanted breeding, simplify management and handling, as well as improve meat quality [6]. Male animals are castrated to avoid high concentrations of androstenone (5α-androst-16-en-3-one), which is a volatile steroid synthesized in boar testes and stored in fat, leading to a urine-like odor in fat and pork [7]. This compound was first identified as the main responsible of the boar taint in 1968 [8] but was later confirmed that it was not the only responsible. In fact, the presence of skatole in fat is also considered a major cause of boar taint [9]. Skatole (3-methyl indole) is a product of the microbial degradation of the amino acid tryptophan, first isolated from boar fat in 1969 [10,11] and leads to a fecal-like odor in fat and meat products [12]. This microbial degradation that produces skatole is only conducted by a small number of bacteria species [13]. This production is increased through the colon and reaches its peak in the distal part of the large intestine [14]. A portion of this skatole is absorbed into the blood and then is carried to the liver, where the cytochrome p450 is able to degrade more than 50% of the amount absorbed, a percentage that is higher in female or castrated animals than in intact male pigs [15]. The skatole that remains in the blood is then deposited in the adipose tissue due to its lipophilic characteristics [14]. A higher presence of skatole in fat is associated with a lower expression of cytochrome P450 2E1 (*CYP2E1*), which led to the conclusion that in pigs *CYP2E1* is negatively correlated with the accumulation of skatole [9].

The concentration of androstenone is closely associated with sexual development [5] and the use of a nutritional approach to modify its value is a controversial topic. Some authors mention no effect [16,17] while others have observed a decrease [18,19,20] namely via induction of hepatic 3β-hydroxysteroid dehydrogenase [21]. However, diet plays a crucial role in modulating skatole production in the gastrointestinal tract [22]. Studies have demonstrated that the amount and type of protein and carbohydrate in the diet can influence the amount of skatole produced [23]. Diets with a high concentration of fermentable carbohydrates, which bypass small intestine digestion, have been found to reduce skatole production. However, the results in the literature regarding these diets are mixed [14]. Some authors have reported that diets rich in sugar beet pulp had no effect on skatole accumulation levels [24], while others have claimed that it leads to a significant reduction in skatole levels [25,26].

This study aimed to investigate the differences in the backfat transcriptome between castrated and intact animals, with the objective of identifying the most expressed genes in each group to further understand the genetic mechanisms that are active when the testes are removed. Additionally, an experimental group of intact animals was fed a high-fiber diet consisting of locally produced legumes, beet pulp and agro-industrial by-products, aiming to reduce boar taint in intact animals as an alternative to surgical castration. To our knowledge, this is an innovative study, since other studies with pigs of European fatty autochthonous breeds finished at these weights are not available.

## 2. Materials and Methods

### 2.1. Animals and Tissue Sampling

Procedures used in this research were approved by the Bioethical Committee for Animal Experimentation (ORBEA) of Évora University (process GD/38814/2020/P1), which meets the European Union Directive 2010/63/EU about the protection of animals used in research.

The design of this study is detailed elsewhere [27]. Briefly, starting in April, a subset of twenty-one pure AL male pigs were raised outdoors in three separate parks, at a stocking density of 100 m^2^ per head, with individual stalls equipped with feeders and water systems. Each park was equipped with a zinc shelter and had some trees and a water pond to help with the high temperatures of summer. Animals were divided evenly into three groups (seven animals per group); one group was composed of surgically castrated animals at an early age (C), one was composed of intact animals (I) and the last one included intact animals that were fed during fattening with an experimental diet (IE). All animals were fed *ad libitum* with commercial diets from ~40 to 130 kg body weight, and during the finishing period, from ~130 to 160 kg (slaughter weight) C and I were fed the same commercial diet, while IE changed to an isoenergetic and isoproteic experimental diet with the addition of locally produced ingredients and agro-industrial by-products. As previously mentioned, these were chosen because they reduce boar taint in intact pigs (see commercial and experimental diet composition in Supplementary Table S1). Diet formulations are proprietary information.

After the finishing period, all animals were slaughtered at ~160 kg, and samples from the subcutaneous fat tissue were collected from the lumbar area, they were vacuum sealed and stored at −80 ºC until used for RNAseq analysis. In the slaughterhouse, weights and yields were registered for carcass, and carcass cuts.

### 2.2. Carcass Traits and Tissue Sampling

At slaughter, carcasses were weighed after evisceration and measures and cuts were obtained from the split left half-carcasses. Backfat thickness was the result of the average from two measures, taken at the 10th rib, and between the last thoracic and first lumbar vertebrae. ‘Zwei punkte’ (ZP) fat and muscle depths were determined, respectively, as the minimal fat (plus rind) depth over the muscle *Gluteus medius*, and the minimal muscle depth between the cranial end of the *Gluteus medius* and the dorsal part of the medullar canal. Carcasses were processed in commercial cuts according to the Portuguese Norm [28] and all those cut’s weights were recorded. From the left-half carcasses samples from *Longissimus lumborum* (LL—loin), and dorsal subcutaneous fat (DSF) were collected, vacuum packaged and frozen (−20 and −80 °C) until analysis.

### 2.3. Muscle and Fat Quality Traits

Moisture from LL and DSF was determined following the ISO-1442 protocol [29]. Total nitrogen from muscle and fat was determined by the Dumas combustion method [30] in a Leco FP-528 Nitrogen/Protein Determinator (Leco Corp., St. Joseph, MI, USA) subsequently calculating crude protein content (N × 6.25). LL and DSF lipids were extracted from the muscles using the Folch method [31]. The fatty acid profiles from LL and DSF were obtained by transesterification into methyl esters [32] and after, the identification and profiling were performed using a Shimadzu GC-MS2010 Plus chromatograph (Kyoto, Japan), equipped with an SP-2560 capillary column (100 m × 0.25 mm I.D., 0.20 μm) (Supelco, Bellefonte, PA, USA). The LL pH after 24 h *post mortem* was determined using a pH meter with a puncture electrode (LoT406-M6-DXK-S7/25, Mettler-Toledo GmbH, Gießen, Germany) [33]. To obtain the LL myoglobin content, heme pigment concentration was first determined with the Hornsey method [34], and then it was multiplied by the factor 0.026 [35]. In order to obtain the LL total collagen content in muscle, first the total hydroxyproline was quantified with the Woessner method [36] and later, this value was multiplied by the factor 7.14 [37].

LL and DSF surface color was determined with a CR-400 colorimeter (Konica Minolta Sensing Europe B.V., Nieuwegein, The Netherlands) equipped with a D-65 illuminant, and in the case of the muscle, after 30 min of blooming. The CIE (Commission Internationale de l’Éclairage) L* (lightness), a* (redness), and b* (yellowness) values were averaged out of six random readings across each sample surface. LL Warner–Bratzler shear force was measured perpendicular to the direction of muscle fibers in cooked rectangular meat sections (1 cm × 1 cm × 3 cm) on a Texture Analyser TA HD Plus (Stable Micro Systems Ltd., Surrey, UK) and a Warner–Bratzler V-shaped shear blade (1.2 mm thick). This method, already described by Martins et al. [38], used an average of the values determined from at least 10 sections of each sample for the statistical analysis.

### 2.4. RNA Extraction

Samples from the dorsal adipose tissue stored at −80 °C were used for the RNA extraction, following a modified version of the PureLink^TM^ RNA Mini Kit from Invitrogen (Thermo Fisher Scientific, Waltham, MA, USA). The modification consists of an additional centrifugation of 10 min after homogenizing the sample with the polytron and taking the supernatant to another tube in order to improve the purity of the extract. RNA purity and quantification were checked with a Quawell NanoDrop Q9000 spectrophotometer (Quawell Technologies, Beijing, China). All samples were checked for integrity using Agilent Bioanalyzer-2100 equipment (Agilent Technologies, Inc., Santa Clara, CA, USA) and all of them passed the quality control test with an average higher than 7 RIN.

### 2.5. Library Preparation

Library preparation and sequencing of the samples were performed at the CNAG institute (Centro Nacional de Análisis Genómico, Barcelona, Spain). A paired-end library was prepared for each sample using TruSeq Stranded mRNA kit (Illumina, Inc., San Diego, CA, USA). Libraries were identified using specific barcoding labels to run in Illumina NovaSeq 6000 instruments (Illumina, Inc., San Diego, CA, USA). Using this protocol reads of 50 bp were obtained, with an average of 50.94 million paired reads per sample.

### 2.6. Bioinformatic Analysis

The obtained Illumina reads were mapped against the *S. scrofa* reference genome Sscrofa11.1 using STAR aligner version 2.7.8a [39] with ENCODE parameters. Annotated genes were quantified with RSEM version 1.3.0 [40] with default parameters, using release 107 of the *S. scrofa* ENSEMBL annotation.

Differential expression analysis was performed with limma v3.42.3 R package, using TMM normalization. The voom function [41] was used to estimate the mean-variance relationship and to compute observation-level weights. The linear model was fitted with the voom-transformed counts and contrasts were extracted. Genes were considered differentially expressed with a *p*-value adjusted <0.05. Functional enrichment analysis was performed with the differentially expressed genes (DEG) using gprofiler2 v0.1.8 [42], based on the ENSEMBL databases as a reference.

### 2.7. RT qPCR Validation Analysis

The technical validation of the RNAseq was performed with genetic material obtained from the 21 animals of the study and 10 genes that were expressed in the three treatment groups: *ACACA*, *ACLY*, *ADIPOQ*, *ELOVL6*, *FASN*, *LEP*, *ME1*, *SCD*, *FABP4* and *IGF1*. Stability was calculated and data was normalized by using the GeNorm software 3.0 [43] and the housekeeping genes: *RPL19*, *HSPCB* and *ACTB*. The normalized data were compared using Pearson to the expression values obtained in the RNAseq analysis to check the concordance correlation coefficient (CCC) [44]. All samples were run in triplicate and no template controls were added to every run.

### 2.8. Statistical Analysis

Results are presented as means ± standard errors (SE). All data were tested for normality by the Shapiro–Wilk test. Statistical analysis was performed by one-way analysis of variance (ANOVA) with the IBM SPSS Statistics software (IBM SPSS Statistics for Windows, v24.0, IBM Corp., Armonk, NY, USA). Differences were considered significant when *p* < 0.05 and *p*-values between 0.05 and 0.10 were considered trends.

## 3. Results and Discussion

### 3.1. Effect of Castration and Diet in Carcass Traits, Meat Quality Traits, and in Fatty Acid Composition

As previously observed [45], castrated animals undergo metabolic regulatory processes that lead to higher accumulation of adipose tissue in subcutaneous, ham, and visceral fat compared to intact pigs. Our study’s findings are consistent with these results, as the C pigs exhibited the highest fat cuts weight due to higher belly and backfat weight, the lowest lean-to-fat cuts ratio, and the thickest average backfat thickness, as shown in Table 1. While there were no statistical differences in carcass weight among the experimental groups, the I animals had lower carcass yield compared to the IE and C groups. This may be due to the fact that the I animals had a numerically lower carcass weight compared to the other two groups. Moreover, the I animals also showed numerically lower weights in fat cuts and ZP fat depth, which could have influenced carcass yield, known to increase with higher fat content in the carcass [46]. Finally, the removal of testes at slaughter did not impact carcass yield in intact pigs, as the testes weight was similar between I and IE pigs (724.6 and 744.4 g, respectively).

**Figure 1:**
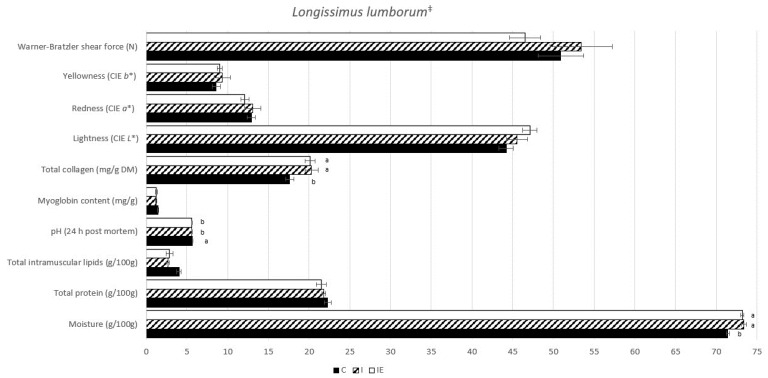
Means of LL quality traits from castrated (C), intact (I) and intact experimental (IE) Alentejano pigs slaughtered at ~160 kg LW. Notes: ^‡^ These data were obtained from a subset of pigs (*n* = 7) used in the study conducted by Martins et al. [27]; ^a,b^ Indicates a significant difference (*p*-value < 0.05).

**Figure 2:**
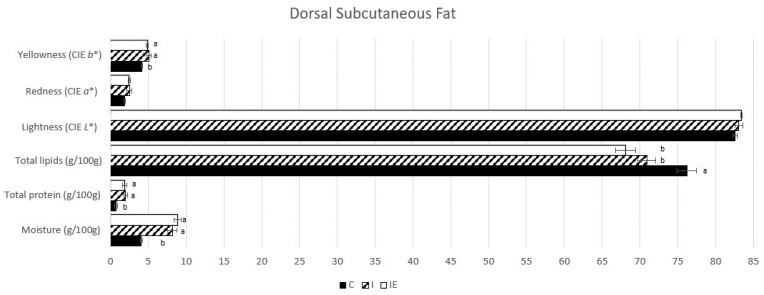
Means of dorsal subcutaneous fat quality traits from castrated (C), intact (I) and intact experimental (IE) Alentejano pigs slaughtered at ~160 kg LW. Notes: ^a,b^ Indicates a significant difference (*p*-value < 0.05).

Meat quality traits were analyzed in the LL muscle and the DSF, as presented in Figure 1 and Figure 2, respectively. In the muscle, differences were observed in C animals, including significantly lower moisture (*p*-value < 0.0001) and total collagen levels (*p*-value = 0.017), as well as the highest total intramuscular lipid content (*p*-value = 0.009). Moreover, the LL pH 24 h *post mortem* was significantly higher (*p*-value = 0.029) in the C animals compared to both intact groups (I and IE). Regarding fat, both intact groups exhibited higher values of moisture (*p*-value < 0.0001), total protein (*p*-value = 0.002), and yellowness (b*) (*p*-value = 0.011). On the other hand, the C animals had higher values of total lipids (*p*-value = 0.001). No significant differences were found among the intact animals (I and IE) in any of the analyzed traits, suggesting that the experimental diet had no effect on meat quality traits.

**Figure 3:**
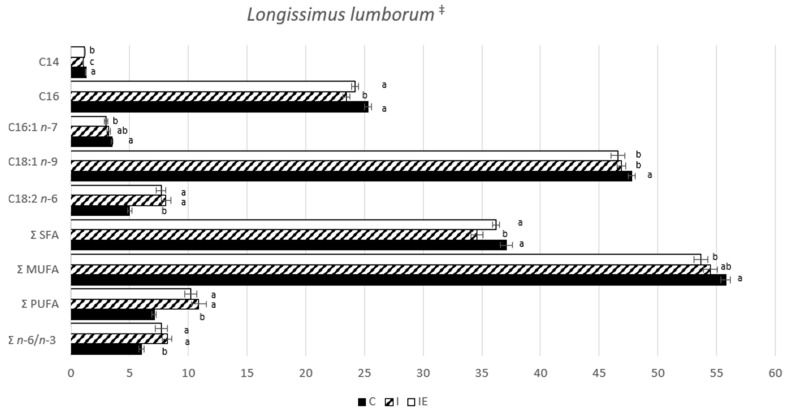
Fatty acid profile (g/100 g) of LL intramuscular lipids from castrated (C), intact (I) and intact experimental (IE) Alentejano pigs slaughtered at ~160 kg LW. Notes: ^‡^ These data were obtained from a subset of pigs (*n* = 7) used in the study conducted by Martins et al. [27]; ^a,b,c^ Indicates a significant difference (*p*-value < 0.05).

**Figure 4:**
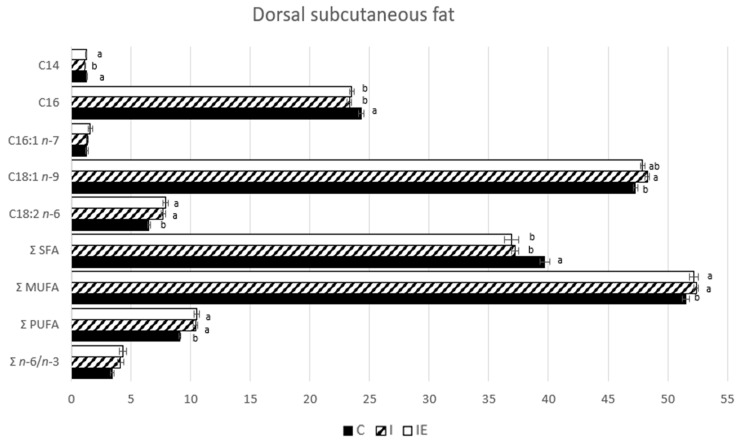
Fatty acid profile (g/100 g) of dorsal subcutaneous fat from castrated (C), intact (I) and intact experimental (IE) Alentejano pigs slaughtered at ~160 kg LW. Notes: ^a,b^ Indicates a significant difference (*p*-value < 0.05).

The fatty acid profile of the LL muscle and the DSF was analyzed, and the results are presented in Figure 3 and Figure 4, respectively. In terms of the muscle, the C animals exhibited the highest levels of myristic acid (C14:0, *p*-value < 0.0001), palmitic acid (C16:0, *p*-value < 0.001), atherogenic index (ATH, *p*-value < 0.0001), and the lowest levels of linoleic acid (C18:2 *n* - 6, *p*-value <0.0001), polyunsaturated fatty acids (PUFA, *p*-value < 0.0001) and *n* − 6 to *n* - 3 ratio (*p*-value = 0.001). These findings suggest that castrated animals have a lower overall deposition of PUFA in muscle tissue and a higher deposition of saturated fatty acids (SFA). The intact animals (I and IE) showed similarities in most traits, but the I animals had a lower saturation index (SAT) compared to the IE ones. This difference was attributed to a slight increase in the proportions of monounsaturated fatty acids (MUFA) in the I pigs, suggesting a subtle modification of the fatty acid profile in the muscle due to the experimental diet. This resulted in a slightly more saturated muscle. These results agree with the numerically higher values of fat cuts, average backfat thickness, ZP fat depth, and intramuscular fat observed in the IE compared to the I pigs. Regarding the DSF, the I animals had lower levels of C:14 (*p*-value = 0.032) than the other two groups, and higher levels of oleic acid (C18:1 *n* - 9, *p*-value = 0.017) compared to the C animals. Unlike the muscle, the IE group showed no differences in C18:1 *n* - 9 compared to the C group and no differences in terms of SFA, MUFA, PUFA or SAT and ATH indexes compared to the I group. Based on these results, it can be suggested that diet has a lesser impact on fat composition compared to muscle. This supports the suggestion that FA synthesis in muscle and fat is independently regulated [47].

### 3.2. Sequencing and Mapping Statistics

An average of 55 million paired-end reads with a size of 50 bp were obtained per sample from the subcutaneous adipose tissue of 21 animals detecting an average of 21,227 genes. Using the reference genome Sscrofa11.1, the paired-end reads were mapped with an alignment rate of 93.4% as an average, which is in line with other studies using the same reference genome [48,49].

### 3.3. Differential Gene Expression Analysis

**Figure 5:**
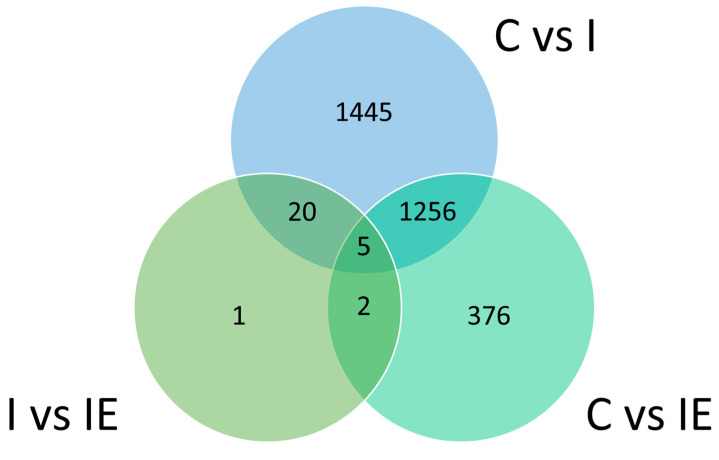
Venn diagram with the common genes between the three comparisons of castrated (C), Intact (I) and Intact experimental (IE) Alentejano pigs.

Since there are three treatment groups, results from the differential analysis of the subcutaneous adipose tissue will be presented in three comparisons: C vs. I, C vs. IE and I vs. IE. From all the detected genes, only those who met the threshold “|log_2_ FC| > 0.58, q < 0.05” were kept as differentially expressed genes (DEG). A total of 2726 DEG were kept when comparing C and I, with 1556 upregulated in C and 1170 upregulated in I. In C vs. IE, 1639 DEG were found with 915 upregulated in C and 724 upregulated in IE. Lastly, in the I vs. IE comparison, only 28 DEG met the threshold, 16 upregulated in I and 12 upregulated in IE. There are several genes in common in the three comparisons represented in Figure 5, 1261 in common between C vs. I and C vs. IE, 25 between C vs. I and I vs. IE, and 7 between I vs. IE and C vs. IE. Finally, there are five genes in common among all three comparisons. All the DEGs detected in the three comparisons can be found in Supplementary Table S2, and the log cpm counts of all the genes analyzed within the limma software are presented in Table S3.

When comparing C to I, the subcutaneous adipose tissue of C pigs showed upregulation of genes related to lipid metabolism, including *LEP*, *SCD*, *FASN* and *ELOVL6*, with differences in expression of more than 2 LogFC. The *LEP* gene encodes the protein leptin, which is secreted by white adipocytes and serves as a circulating signal for nutritional status. Leptin plays a crucial role in regulating food intake, energy expenditure, and whole-body energy balance. Its expression and secretion are strongly correlated with body fat mass and adipocyte size [50,51]. The increase in circulating leptin is a biomarker for leptin resistance [52], and is in line with the significantly higher average total feed consumption of C animals, which presented a higher *LEP* expression, and with the significantly lower average total feed rejection. These observations suggest a potential decrease in the capacity of leptin to suppress appetite in C animals. In the closely related Iberian pig, a single nucleotide polymorphism (SNP) was identified in the *LEPR* gene, which is associated with leptin resistance. This SNP was fixed in that breed and it was stated that could be one of the determinants of the obese phenotype characteristic of the Iberian breed [53]. It is possible that this SNP may also be present in the AL breed, contributing to leptin resistance in this breed. Furthermore, the increase in *LEP* activity in C animals could be linked to testosterone production. A study in humans found that individuals with low serum testosterone levels had significantly higher levels of leptin in their blood [54]. Overall, these findings highlight the upregulation of genes related to lipid metabolism, particularly leptin, in subcutaneous adipose tissue of C pigs, and suggest possible links to leptin resistance, breed-specific genetic factors, and hormonal influences on leptin activity.

The *SCD* or Stearoyl-CoA desaturase gene is responsible for the biosynthesis of MUFA, particularly C18:1 *n* - 9, through the desaturation of stearic acid (C18:0) [55]. This gene is also associated with the total fat content in muscle [56] and has previously been found to be upregulated in castrated AL pigs compared to Bísaro pigs [48], a Celtic-type breed [57]. The results obtained in this study agree with the aforementioned findings, as the C animals presented significantly higher proportions of C18:1 *n* - 9 in the total intramuscular lipids. This suggests a higher C18:1 *n* - 9 and IMF biosynthesis in the muscle of C animals. However, these differences were not observed in the DSF. This discrepancy suggests an independently regulated FA synthesis in muscle and fat [47] and/or could be attributed to the differences in percentage values, as the C animals showed significantly higher levels of total lipids. In this case, although the total value of C18:1 *n* - 9 in C animals could be higher than in the other two groups, the increases in other fatty acids might be masking this difference. Additionally, since MUFAs such as C18:1 *n −* 9 are preferred as a fuel source for exercise in pigs [58], their oxidation could also have contributed to the lower levels of this MUFA observed in the muscle of the more exercised intact pigs [27].

The *FASN* gene encodes the Fatty Acid Synthase, a multi-enzyme protein involved in the *de novo* synthesis of C16:0 from acetyl-CoA and malonyl-CoA. Additionally, it plays a role in the synthesis of long-chain SFAs [59,60]. Previous studies have investigated the relationship between higher expression of *FASN* and an increased risk of obesity due to the accumulation of triacylglycerols in humans [61]. Similar effects have been observed in pigs, where a lower *FASN* expression has been associated with lower fat accumulation [62,63,64]. In line with these findings, the C animals in this study presented higher expressions of *FASN* and, as a result, they had significantly more adipose tissue and a higher proportion of C16:0 in both LL and DSF. These observations support the studied effects of the *FASN* gene in castrated AL pigs when compared to intact animals.

The *ELOVL6* gene belongs to the fatty acid elongases family, and they use malonyl-CoA as a 2-carbon donor in the first and rate-limiting step of fatty acid elongation [65]. It is located in a genomic region where a QTL is affecting C16:0, and palmitoleic acid (C16:1 *n* - 7) composition, and studies on its polymorphism have suggested that *ELOVL6* may regulate the overall balance of fatty acids composition [66]. Several SNPs in the *ELOVL6* gene have been found to be strongly associated with the content of C14:0, C16:0, and C16:1 *n* - 7 in IMF and backfat in pigs [67]. These results are fully aligned with the results of our study, where the C pigs exhibited the highest levels of C14:0, C16:0, C16:1 *n* - 7 and total intramuscular lipids in the LL, and of C14:0, C16:0, and total lipids in DSF in C pigs. This coincided with the significantly higher expression of the ELOVL6 gene in these animals. These results suggest that genetic variations in ELOVL6 may contribute to modulating the fatty acid profiles in AL pigs through selective breeding, providing potential targets for future studies. Finally, the lower proportion of PUFA observed in the C pigs could be associated with the significantly higher expression of *ELOVL6* and *FASN* in the adipose tissue [68]. As these two genes are involved in the *de novo* synthesis of SFA and MUFA, the overall percentage of PUFA, from dietary origin, would be reduced accordingly.

In the subcutaneous adipose tissue of C animals, we observed high expression of genes related to collagen metabolism, including *COL8A1*, *COL8A2* and *COL6A6*. However, these genes were not found to be associated with any of the studied carcass, growth and meat quality traits. On the other hand, in the I animals, a different group of genes related to collagen metabolism was differentially expressed, including *COL1A1*, *COL1A2*, *COL4A3* and *COL4A6*. These genes are involved in the synthesis of collagen found in most connective tissues, and more abundant in structures such as bone, cornea, dermis, and tendon. *COL1A1* and *COL1A2* genes are associated with the synthesis of type I collagen fibers, which consist of two pro-α1(I) (*COL1A1*) chains and one pro-α2(I) (*COL1A2*) chain, and are involved in collagen fibril organization and biosynthesis [69]. It has been suggested that the downregulation of collagen precursors can lead to a reduction in the total collagen content in the skeletal muscle [70]. Considering that these genes affect basal collagen metabolism, their downregulation could probably improve chewiness in muscles like the LL [71]. Although our study found that C animals, with the lower expression of *COL1A1* and *COL1A2*, also had significantly lower values of total collagen in LL muscle, the Warner–Bratzler analysis did not reveal significant differences among the experimental groups. This suggests that while there may be an impact on collagen metabolism, it did not translate into noticeable differences in meat tenderness as assessed by the Warner–Bratzler test.

In the subcutaneous adipose tissue from I pigs, the most highly expressed genes are primarily related to muscular activity, i.e., *MYH1*, *ACTA1*, *TNNT3*, *MYL1*, and *ACTN3*. Among these genes, Myosin Heavy Chain 1 (*MYH1*) was the most expressed gene, with a LogFC of 8 (adjusted *p*-value < 0.05). Myosin is composed of both light chains and heavy chains, and *MYH1* specifically encodes the heavy chain IIx, which along with IIa and IIb are considered the “fast” isoforms, also referred to as “fast twitch” or “white muscle” [72,73]. However, IIx is classified as an intermediate or fast oxidative type [74]. *MYH1* is known to play a role in skeletal muscle contraction and is involved in the transition between slow-to-fast and fast-to-slow muscle fibers [75]. In a mouse experiment, it was found that ectopic overexpression of *MYH1* led to a shift in muscle fiber composition towards slower muscle fibers [75]. Muscle fiber composition is closely related to meat quality, and a higher percentage of intermediate fibers (IIa and IIx) is associated with reduced *post mortem* metabolism rates [76]. Studies have suggested that pigs with higher numbers of type I and intermediate fibers have lower drip loss, lower L* value, and higher muscle pH [76]. However, the results of our study do not fully align with these findings. Contrary to expectations, C animals had the highest pH in the LL muscle (*p*-value = 0.029). Regarding water-holding capacity, muscles with a higher content of intermediate fibers are expected to have lower moisture content, and higher thawing/cooking losses [77]. In our data, the LL and DSF from C animals had lower moisture content (*p*-value < 0.001) and, although not statistically significant possibly due to large standard errors, had higher thawing losses (C: 1.49, I: 0.90, IE: 0.95; *p*-value = 0.227). These contradictory results could be attributed to the total intramuscular lipids content in C animals. It is known that fat can affect water-holding capacity and increase losses, as observed in lambs [78]. Additionally, higher intramuscular fat content is associated with greater muscle color intensity [79]. Considering this, the influence of fiber type may have been masked by the effect of intramuscular fat in C pigs, making the effects of muscle fibers less significant.

In subcutaneous adipose tissue from I animals, another highly expressed gene related to muscular activity is *ACTA1*, with a LogFC of 6.59. The protein encoded by the *ACTA1* gene corresponds to the monomeric form of actin filaments, which is a major component of the contractile apparatus in skeletal muscle [80]. Previous studies have shown that *ACTA1* levels are positively correlated with the synthesis of new muscle fiber proteins and overall muscle growth [81,82]. Since *ACTA1* is a gene involved in basal metabolism, we can hypothesize that its expression follows a similar profile in the rest of the tissues. In our study, I (and IE) animals presented a significantly higher lean-to-fat cuts ratio, due to their higher commercial yield and lower fat cut weight and ZP fat depth compared to C animals. This indicates that intact animals were leaner than castrated ones. Furthermore, a significantly lower blood urea level was also detected in I animals at 160 kg LW [27], which has been previously associated with more efficient nitrogen utilization and increased lean tissue [83]. These findings support the leaner profile of I (and IE) animals. In a study comparing Yorkshire pigs and Shaziling pigs, a heavy Chinese breed [81], the leaner and more muscular breed (Yorkshire) also presented the highest *ACTA1* expression values. Therefore, the higher expression of *ACTA1* in intact animals may be one of the factors contributing to their leaner and less fatty phenotype compared to C ones.

When comparing subcutaneous adipose tissue from C to IE, we found overexpressed genes related to lipid and collagen metabolisms in C animals, while DEGs mainly related to collagen metabolism were identified in IE animals. Interestingly, many of the genes upregulated in C animals in relation to lipid metabolism were also found to be differentially expressed in the C vs. I comparison, including *LEP*, *ME1*, *FABP4* and *ELOVL6.* These shared genes exhibited similar expression patterns in IE as in the I animals, suggesting that the differences observed may be primarily attributed to surgical castration rather than diet-related effects.

Malic enzyme 1 (*ME1*) is a key enzyme involved in the tricarboxylate shuttle, which provides NADPH and acetyl-CoA for fatty acid biosynthesis [84]. Previous studies in AL pigs have linked higher expressions of ME1 to overall increased lipid synthesis [48]. These results align with the ones in the current study, as C animals also presented significantly higher overall lipid deposition, including backfat, belly, and intramuscular fat in LL, as well as the highest lipid content in DSF. *ME1* overexpression has also been associated with a higher proportion of C16:0 in certain local strains of Iberian pigs [59], which is consistent with our study, where C animals exhibited the highest C16:0 proportion in both DSF and LL. These results suggest that C animals may have a higher metabolic capacity for lipid synthesis.

Fatty acid binding protein 4 (*FABP4*) is predominantly expressed in adipose tissue and plays a major role in lipid metabolism and adipocyte homeostasis. It interacts with peroxisome proliferator-activated receptors, binding to hormone-sensitive lipase and facilitating lipid transport and utilization [85,86,87]. Previous studies have reported that *FABP4* falls within a QTL interval associated with marbling in bovine populations [85]. It has also been associated with differences in IMF content in Duroc pig populations, with no significant effects on backfat thickness [88]. Our results in AL pigs are in line with these previous studies, as C animals presented the highest lipid content in DSF and significantly higher intramuscular lipid values in LL. Additionally, blood levels of triacylglycerols analyzed on these pigs and reported by [27], were also significantly higher in C pigs at ~160 kg LW, further supporting the notion of increased lipid metabolism in C animals, particularly in the finishing period.

The same genes related to collagen metabolism as seen in the subcutaneous adipose tissue of C vs. I showed similar expression patterns in the C vs. IE comparison, with *COL8A1* and *COL6A6* upregulated in C, and *COL1A1* and *COL1A2* upregulated in IE. These results are consistent with the observations in I animals, as there were no significant differences in total collagen content or the results of the Warner–Bratzler test between I and IE groups.

The comparison between subcutaneous adipose tissue of I vs. IE animals did not reveal any DEGs related to muscular, lipid, or collagen metabolism. This suggests that the experimental diet did not significantly influence the expression of genes involved in these metabolisms. Therefore, the observed differences in gene expression in the other two comparisons (C vs. I and C vs. IE) are likely attributed to the surgical removal of the testes rather than the experimental diet. These findings indicate that the experimental diet had no major negative effects on meat quality issues.

As reported by Martins et al. [27], although overall lower than expected, the content of androstenone in neck subcutaneous fat was significantly lower in C pigs than in I and IE pigs, while skatole content was not affected by experimental treatments. Skatole metabolism is modulated by CYP enzymes of the p450 cytochrome in the hepatic tissues [15]. Among the genes related to boar taint in this cytochrome, the main gene involved is CYP2E1. This gene expression in the liver is negatively correlated with skatole accumulation [9]. In this study, *CYP2E1* was not found to be differentially expressed in fat tissue in any of the comparisons. Interestingly, other genes of the cytochrome p450 family, namely *CYP1A1* and *CYP2A19*, showed significant differential expression across all comparisons. *CYP1A1* was significantly more highly expressed in both intact groups (I and IE) compared to the C group, while *CYP2A19* was more highly expressed in IE than in I animals. These findings challenge the previous notion that *CYP2E1* is the main gene involved in skatole degradation, which might not have been adequately tested, as suggested by Wiercinska et al. [89]. From the studies of Friis [90], it was deduced that a decreased activity of *CYP2E1* led to a decrease in the clearance of skatole in some pigs. This was based on the decreased ability to metabolize chlorzoxazone (CLZ), a substrate used as a probe for *CYP2E1*. Although this is correct, more recent studies [91] have questioned the specificity of CLZ for *CYP2E1*, and it was found that porcine *CYP2A19* and *CYP1A1* also contributed to CLZ hydroxylation [92]. In this way, the role of these genes in the skatole degradation was studied [89] and it was found that *CYP2A19* was the primary isoform responsible for the formation of 6-hydroxyskatole, the main metabolite that is found in the plasma of pigs that efficiently metabolize and clear skatole. Furthermore, *CYP1A1* was also identified as capable of producing one of the most abundant skatole metabolites, 3-methyloxyindole. In this way, the experimental diet could have induced an increase in skatole degradation due to the higher expression of the *CYP2A19* gene in the IE group. However, the fact that *CYP1A1* was mostly expressed in intact animals (I and IE) when compared to C, disagrees with other studies that claim that castrated animals have higher gene expression of *CYP1A* subfamily enzymes [93,94]. Although interesting, the magnitude of these effects seems not to have been sufficient to show practical effects, since neck fat skatole content was not significantly different between I and IE pigs, fed a commercial and the experimental diets, respectively [27]. These results need confirmation by future transcriptomic studies, namely in hepatic tissues.

### 3.4. Validation Analysis

The results obtained from RNAseq analysis were consistent with the ones obtained in the qPCR analysis, as shown in Table 2, and primer designing information for the validation test, the raw CT values and normalization factors are found in Table S4**.** The slight variations in gene expression between the two methods can be attributed to the differences in accuracy and sensitivity of each technique. Despite these variations, the Concordance Correlation Coefficient (CCC) of all three comparisons is above 0.7, indicating a strong correlation between the two methods.

### 3.5. Functional Analysis

The functional analysis identifies biological pathways that are enriched using the DEGs more than would be expected by chance [95]. Since there are three comparisons, a separate functional analysis will be presented for each of them. The corresponding data can be found in Supplementary Table S5.

In the comparison between C and I, a total of 725 enriched pathways were identified with a *p*-value of less than 0.05 as a combination of Gene Ontology (GO), Biological Processes (BP), Cellular Component (CC), Molecular Function (MF), and KEGG enrichment analysis. There are two clearly defined groups, one with pathways related to lipid metabolism or fatty acid composition, and the other related to muscular activity and development. In the first group, several Biological Processes pathways were identified, including “lipid metabolic process” (GO:0006629), “carboxylic acid metabolic process” (GO:0019752), “fatty acid metabolic process” (GO:0006631) and “fatty acid oxidation” (GO:0019395). In the Cellular Component pathways, we found “lipid droplet” (GO:0005811), in the Molecular Function pathways we found “lipid binding” (GO:0008289) and in the KEGG enrichment analysis we found “Fatty acid metabolism” (KEGG:01212) among many others. These data support the results obtained from the differential analysis, confirming that the removal of the testes can influence the expression of genes involved in lipid metabolism. In the group related to muscular activity, several Biological Processes pathways were identified, including “actin filament-based process” (GO:0030029), “actin filament organization” (GO:0007015) and “growth” (GO:0040007). The Cellular Component pathway “actin filament” (GO:0005884) and Molecular Function pathway “actin binding” (GO:0003779) were also enriched, among others. Similar to the previous group, these pathways are in line with the results from the differential analysis, confirming that intact and castrated animals exhibit differential expression in genes associated with growth and/or muscular activity.

In the comparison C vs. IE, a total of 462 enriched pathways with a *p*-value of less than 0.05 were identified. Interestingly, these pathways largely overlap with the ones in the previous comparison (C vs. I). For the first group related to lipid metabolism, pathways such as “lipid metabolic process” and “lipid droplet” were again enriched. Similarly, the second group associated with muscular activity included pathways like “actin filament-based process” and “growth”. These findings suggest that the observed differences in gene expression related to lipid metabolism and muscular activity are primarily due to castration, and there appear to be no noticeable effects of the diet on these pathways.

In the comparison I vs. IE, only two enriched pathways were identified, one Cellular Component pathway related to “mitochondrion” (GO:0005739) and a KEGG pathway named “Metabolic pathways” (KEGG:01100). However, these pathways do not provide significant evidence of specific biological processes related to the diet. Based on these results, it is not possible to conclude that the diet has a significant impact on the activation or inactivation of pathways related to lipid metabolism or muscular activity. Therefore, it is likely that the pathways reported in the previous comparisons are primarily influenced by the removal of the testis.

## 4. Conclusions

Overall, the phenotypic differences in carcass and meat quality traits were mainly due to the surgical removal of the testis. The intact animals (I and IE) exhibited a leaner phenotype compared to the C pigs, which presented a fattier profile. These results align with the transcriptomic analysis of the subcutaneous fat; intact animals showed increased expression of genes associated with muscular activity (*ACTA1*, *MYH1*) and collagen metabolism (*COL1A1*, *COL1A2*), while C animals exhibited higher expression of lipogenic genes (*ME1*, *ELOVL6*, *FASN*, *SCD*).

Regarding the diets, no significant effects on meat quality traits were observed, except for a potential subtle alteration in the muscle fatty acid profile, resulting in a lower saturation and higher polyunsaturation level in the tissues of intact pigs. However, it is worth noting that the experimental diet did contribute to a reduction in stress, as indicated by significantly lower cortisol levels at the end of the finishing period (~160 kg LW) [27].

Considering the higher expression levels of *CYP2A19* in the adipose tissue of pigs from the IE group, the experimental diet could be associated with an increased skatole metabolism in the liver. However, these results need to be confirmed, namely through hepatic tissue transcriptomic studies. Furthermore, in practical terms, this potential did not translate into significant changes in skatole fat content in I and IE pigs in this study.

In summary, our study provides comprehensive insights into the phenotypic differences, meat quality traits, and gene expression patterns in intact AL pigs. The results highlight the impact of castration on adipose tissue accumulation, fatty acid profile, and pathway activation. Further research with larger sample sizes and different experimental diet formulations, namely by changing the ingredients percentage and/or choosing new ingredients, is needed to validate these findings and explore alternative strategies for boar taint reduction.

## Figures and Tables

**Table 1:** Means of carcass, cut traits and growth data from castrated (C), intact (I) and intact experimental (IE) Alentejano pigs slaughtered at ~160 kg LW *.

Trait	C	I	IE	*p*-Value
Hot carcass weight (kg)	125.0	120.3	124.6	0.163
Carcass yield (%)	78.8 ^a^	75.7 ^b^	77.8 ^a^	<0.001
Commercial yield (kg) ^1^	29.3	29.5	30.7	0.133
Untrimmed shoulder (kg)	10.6 ^c^	11.7 ^b^	12.6 ^a^	0.012
Loin (kg)	2.95	2.96	2.85	0.651
Untrimmed ham (kg)	15.5 ^a^	14.5 ^b^	15.0 ^ab^	0.029
Tenderloin (g)	315.4	342.3	312.9	0.281
Fat cuts (kg) ^2^	17.9 ^a^	15.0 ^b^	15.3 ^b^	0.005
Belly (kg)	7.7 ^a^	5.9 ^b^	6.7 ^b^	0.014
Backfat (kg)	10.2 ^a^	9.0 ^b^	8.7 ^b^	0.019
Lean to fat cuts ratio	1.64 ^b^	1.98 ^a^	2.01 ^a^	<0.001
Average backfat thickness (mm) ^3^	69.4 ^a^	51.1 ^b^	52.4 ^b^	<0.001
Fatness: ZP fat depth (mm) ^4^	64.3 ^a^	47.9 ^b^	49.4 ^b^	<0.001
Average total feed consumption (kg)	544.0 ^a^	486.1 ^b^	454.1 ^c^	<0.001
Average total feed rejection (kg)	2.36 ^b^	18.05 ^a^	1.76 ^b^	<0.001

* These data were obtained from a subset of pigs (*n* = 7) used in the study conducted by Martins et al. [27]; ^1^ Sum of the untrimmed shoulder, untrimmed ham, loin, and tenderloin cuts; ^2^ Sum of the belly and backfat cuts; ^3^ Average of measurements taken at the 10th rib level, and last thoracic and first lumbar vertebrae (last rib level); ^4^ Minimal fat depth (including rind) over the muscle *Gluteus medius*; ^a,b,c^ Values in the same row with different superscript letters are significantly different (*p* < 0.05).

**Table 2:** Validation test with the log fold changes of the results from qPCR and RNAseq and the estimated CCC values.

Gene	C vs. I		C vs. IE		I vs. IE	
	RNAseq	qPCR	RNAseq	qPCR	RNAseq	qPCR
*ACACA*	1.001	1.432	0.595	0.803	−0.406	−0.629
*ACLY*	0.837	1.203	0.816	0.964	−0.021	−0.239
*ADIPOQ*	1.076	0.834	0.209	0.198	−0.868	−0.636
*ELOVL6*	1.987	2.120	1.033	0.872	−0.953	−1.248
*FASN*	2.021	1.033	1.092	0.637	−0.929	−0.395
*LEP*	2.486	2.487	1.817	1.455	−0.669	−1.032
*ME1*	1.798	1.542	1.312	1.371	−0.486	−0.171
*SCD*	2.241	2.532	1.026	1.099	−1.215	−1.433
*FABP4*	0.190	−0.439	1.273	−0.030	0.470	0.409
*IGF1*	−0.378	0.006	0.357	0.710	−0.080	−0.716
	Concordance Correlation Coefficient					
	C vs. I		C vs. IE		I vs. IE	
CCC	0.876		0.714		0.767	

## Data Availability

Data will not be shared, due to privacy restrictions.

## Supplementary Materials

The following supporting information can be downloaded at: https://www.mdpi.com/article/10.3390/genes14091722/s1; Table S1: Composition of commercial and experimental diets; Table S2: Differentially Expressed Genes; Table S3: Log cpm counts; Table S4: Primer design and raw CT values; Table S5: Enrichment Pathways analysis.
