# Supplementary material for: Transcriptomic Profiling of Subcutaneous Backfat in Castrated and Intact Alentejano Pigs Finished Outdoors with Commercial and Fiber-Rich Diets
Source: Genes (Basel). 2023 Aug 29;14(9):1722. doi: 10.3390/genes14091722 (PMC10531178; doi:10.3390/genes14091722)
Supplement: Supplementary file 1 [file genes-14-01722-s001.zip › Table S1-Diet composition.doc]

**^Table S1.^** ^Composition of the commercial and experimental diets consumed by Alentejano pigs slaughtered at ~160 kg LW^

|  | **Growing diet A^1^** | **Growing diet B^2^** | **Fattening diet^3^** | **Experimental diet^4^** |
| --- | --- | --- | --- | --- |
|  | (g/100 g) | | | |
| Dry matter (DM) | 90.7 | 91.8 | 90.8 | 90.5 |
|  | (g/100 g DM) | | | |
| Total ashes | 7.6 | 7.1 | 8.1 | 8.2 |
| Crude protein (N x 6.25) | 16.8 | 16.0 | 13.8 | 13.8 |
| Total fiber^5^ | 17.4 | 23.6 | 26.9 | 40.2 |
| Insoluble fiber^5^ | 6.1 | 13.1 | 15.1 | 28.0 |
| Neutral detergent fiber (NDF) | 24.1 | 25.3 | 22.6 | 29.9 |
| Acid detergent fiber (ADF) | 7.3 | 11.1 | 9.4 | 13.2 |
| Acid detergent lignin (ADL) | 0.74 | 1.59 | 1.64 | 0.90 |
| Hemicellulose^6^ | 16.8 | 14.2 | 13.2 | 16.7 |
| Cellulose^7^ | 5.5 | 9.1 | 6.5 | 10.4 |
| Total sugars^8^ | 3.5 | 3.1 | 3.0 | 1.88 |
| Total starch^9^ | 47.0 | 51.8 | 45.5 | 40.1 |
| Total lipids | 3.0 | 4.0 | 6.8 | 6.2 |
| Palmitic acid (C16:0) | 0.44 | 0.45 | 0.66 | 0.66 |
| Oleic acid (C18:1 *n*-9) | 1.88 | 3.03 | 4.42 | 5.41 |
| Linoleic acid (C18:2 *n*-6) | 1.33 | 1.01 | 1.66 | 1.55 |
| Saturated fatty acids | 0.44 | 0.56 | 0.88 | 0.88 |
| Monounsaturated fatty acids | 1.88 | 3.03 | 4.42 | 5.41 |
| Polyunsaturated fatty acids | 1.33 | 1.01 | 1.77 | 1.66 |
| Σ *n*-3 | 0.09 | 0.10 | 0.14 | 0.15 |
| Σ *n*-6 | 1.20 | 0.94 | 1.46 | 1.39 |
|  | (MJ/kg) | | | |
| Digestible energy^10^ | 13.6 | 13.2 | 13.2 | 13.1 |
| Notes: ^1^Fed from 40 to 60 kg LW to all experimental groups; ^2^Fed from 60 to 120 kg to all experimental groups; ^3^Fed from 120 to 160 kg to C and I group and from 120 to 130 kg to IExp group; ^4^Fed from 130 to 160 kg to IExp group; ^5^[1] method 991.43; ^6^Hemicellulose = NDF-ADF; ^7^[2]; ^8^[1] method 982.14; ^9^[3]; ^10^[4]. | | | | |

**References:**

1.	AOAC. *Official Methods of Analysis of AOAC International*, 18th ed.; AOAC: Gaithersburg, MD, 2006.

2.	ISO-6865. Animal feeding stuffs - Determination of crude fibre content - Method with intermediate filtration. **2000**, *ISO 6865*, 10.

3.	ISO-6493. Animal feeding stuffs - Determination of starch content - Polarimetric method. **2000**, *ISO 6493*, 10.

4.	Noblet, J.; Fortune, H.; Dupire, C.; Dubois, S. Digestible, metabolizable and net energy values of 13 feedstuffs for growing pigs: effect of energy system. *Animal Feed Science and Technology* **1993**, *42*, 131 - 149.
